# No difference in Oxford Knee Score between medial and lateral unicompartmental knee arthroplasty after two years of follow-up: a clinical trial

**DOI:** 10.1186/s40634-023-00704-x

**Published:** 2023-12-08

**Authors:** Filippo Migliorini, Federico Cocconi, Julia Prinz, Nicola Ursino, Laura Mangiavini, Riccardo D’Ambrosi

**Affiliations:** 1 Department of Orthopaedics, Trauma, and Reconstructive Surgery, RWTH University Medical Centre, Pauwelsstraße 30, 52074 Aachen, Germany; 2 Department of Orthopaedics and Trauma Surgery, Academic Hospital of Bolzano (SABES-ASDAA), Teaching Hospital of the Paracelsus Medical Unviersity, Bolzano, 39100 Italy; 3 Department of Ophthalmology, RWTH University Medical Centre, 52074 Aachen, Germany; 4 CASCO Department, IRCCS Ospedale Galeazzi - Sant’Ambrogio, Milan, Italy; 5 EUORR Department, IRCCS Ospedale Galeazzi - Sant’Ambrogio, Milan, Italy; 6 Department of Biomedical Sciences for Health, University of Milan, Milan, Italy

**Keywords:** Unicompartmental knee arthroplasty, UKA, Oxford Knee Score, Medial, Lateral

## Abstract

**Purpose:**

In patients with monocompartmental knee osteoarthritis, unicompartmental knee arthroplasty (UKA) can be performed. This study compared the medial versus lateral UKA in patients with monocompartimental knee arthroplasty. It was hypothesised that both implants achieve a similar outcome in OKS.

**Methods:**

The UKAs were fixed-bearing medial PPK (Zimmer-Biomet, Warsaw, Indiana, USA) and fixed-bearing lateral Zuk (Lima Corporate, Udine, Italy). An intraarticular drain was placed and removed on the first postoperative day. Enoxaparin sodium 4000 units subcutaneously daily for 45 days was used as thromboembolic prophylaxis. The Italian version of the OKS was used for the clinical assessment. The following complications were also recorded: anterior knee pain, infection and revision surgeries.

**Results:**

Data from 203 patients were collected. The mean age of the patients was 68.9 ± 6.7 years and the mean BMI was 28.1 ± 4.1 kg/m^2^. The mean OKS on admission was 22.1 ± 4.5 points. On admission, women, patients older than 70 years, and those with a BMI lower than 28 kg/m^2^ who underwent lateral UKA evidenced lower OKS. At the last follow-up, 26.7 and 26.9 months for the lateral and medial UKA, respectively, no between groups difference in OKS was evidenced. No patients experienced complications.

**Conclusion:**

Medial and lateral UKA achieve similar outcomes in OKS at a minimum of two years of follow-up.

## Introduction

Osteoarthritis (OA) of the knee is common [39, 57]. Total knee arthroplasty (TKA) is considered the gold standard treatment for end-stage OA of the knee [34–36, 44, 45]. The prevalence of isolated lateral and medial compartment OA is about 7.5% and 25%, respectively [37, 46, 48]. In 1989, Kozinn and Scott highlighted the indications for UKA: stable anterior cruciate ligament, varus deformity lower than 5°, range of motion greater than 90° without flexion contracture, and body mass index (BMI) lower than 30 kg/m^2^ [25]. However, these indications are often considered obsolete [1, 30]. In contrast to TKA, UKA spares the cruciate ligaments and all structures of the contralateral joint compartment [13, 38, 53]. In the last decades, these indications have become outdated, and UKA has been performed in unconventional settings. Indeed, anterior cruciate deficiency and patellar osteoarthritis represent no absolute contraindication [17, 20, 21, 50]. Compared to TKA, UKA is associated with lower intraoperative blood loss, faster recovery, better functional outcomes, and a greater postoperative range of motion [19, 23, 27, 28, 31, 41, 47]. However, approximately 20% of UKA patients undergo revision arthroplasty to TKA [49, 51]. The medial UKA is more commonly performed than the lateral UKA, and in total arthroplasty, a medial parapatellar approach is the standard surgical access. This could negatively impact surgery on the lateral side, which might also necessitate a longer learning curve. Previous studies which compared medial versus lateral UKA found no difference in patient-reported outcome measures (PROMs) [2, 18, 29, 32, 37, 40, 52, 58]. To the best of our knowledge, clinical investigations which compared the outcomes of medial versus lateral UKA in Oxford Knee Score (OKS) are missing. Being the OKS one of the most used PROM for clinical assessment, it is important to investigate whether medial and lateral UKA could exert a difference on it. Therefore, a clinical trial was conducted. The outcomes of interest were to compare the Oxford Knee Score and the rate of complications between the two implants. It was hypothesised that both implants achieve a similar outcome in OKS.

## Methods

### Study protocol

The present study was conducted following the Strengthening the Reporting of Observational Studies in Epidemiology (STROBE) statement [9]. All procedures were conducted in accordance with the standards highlighted in the 1964 Helsinki Declaration and its later amendments. Written informed consent was obtained from all the participants. The present study was approved by the Ethics Committee of the San Raffaele University Hospital of Milan, Italy (CE 236/2017).

### Eligibility criteria

The inclusion criteria were: isolated monocompartmental symptomatic OA stage III to IV according to the Kellgren-Lawrence classification [24], anterior cruciate, medial and lateral collateral ligaments functionally intact, as confirmed by magnetic resonance imaging (MRI) and clinical examination, a range of motion (ROM) of at least 90°, patients able to understand the nature of the study. The exclusion criteria were: previous surgery (except arthroscopic meniscectomy), lower limb axial deformity, peripheric neuropathy or severe arterial disease or presence of ulcers, and any uncontrolled acute blood abnormalities.

### Surgical procedures and rehabilitation

Surgery was performed by one author in a highly standardized fashion at the CASCO Department of the IRCCS Ospedale Galeazzi Sant’Ambrogio, Milan, Italy between September 2018 and January 2021. The implants were fixed-bearing medial PPK (Zimmer-Biomet, Warsaw, Indiana, USA) and fixed-bearing lateral Zuk (Lima Corporate, Udine, Italy). All patients were placed in a supine position on a standard operating table under spinal study. A standard medial or lateral parapatellar approach was used. Inspection of the patellofemoral and medial/lateral compartments was performed. All components were cemented using Refobacin Bone Cement R (Zimmer Biomet, Warsaw, Indiana, USA). An intraarticular drain was placed and removed on the first postoperative day. Enoxaparin sodium 4000 units subcutaneously daily for 45 days was used as thromboembolic prophylaxis. The postoperative protocol was conducted following a previous report [12]. Briefly, both the patient groups followed the same study protocol involving passive mobilisation from day one after the surgery. From day two, they started an active progressive mobilisation of the joint and assisted walking with two crutches. According to each patient’s capability, a gradual increase in the load during walking was recommended, continuing with isometric muscle toning exercises until the total abandonment of walking aids.

### Clinical assessment

The clinical assessment was conducted by two independent clinicians who were not involved in the clinical management of patients. The Italian version of the OKS was used for the clinical assessment [43]. The OKS is a simple patient-reported outcome measure based on a 12-question Likert-like on function, activities of daily living, and pain over the preceding four weeks and demonstrated validity and simple administration [11, 14, 33, 42]. Each question has four possible answers. The final result ranges from 0 (poorest function) to 48 (maximal function). The following complications were also recorded: anterior knee pain, infection and revision surgeries.

### Power analysis

An estimated sample of 71 subjects for each group was required to compare OKS between medial and lateral UKA position with a two-sided Wilcoxon-Mann Whitney test, given an index mean difference of 5, a standard deviation of 8 for both groups, a 5% alpha, an 95% power. This sample had also a 99% power to detect a difference between pre- and post-operative values with a one-sided Wilcoxon signed-rank test, assuming a mean difference of 5, a standard deviation of 8 for both groups, and a 2.5% alpha. Additional subjects were recruited to ensure statistical significance in case of adverse events.

### Statistical analysis

The statistical analyses were conducted by the main study (F.M.) using the software IBM SPSS version 25. For continuous variables, the mean and standard deviation were used. For the comparisons, the mean difference (MD) effect measure and standard error (SE) were adopted. A 95% confidence interval was set as a standard. The unpaired two-tailed t-test was used, with values of *P* > 0.05 considered statistically significant.

## Results

### Patient recruitment

**Fig. 1:**
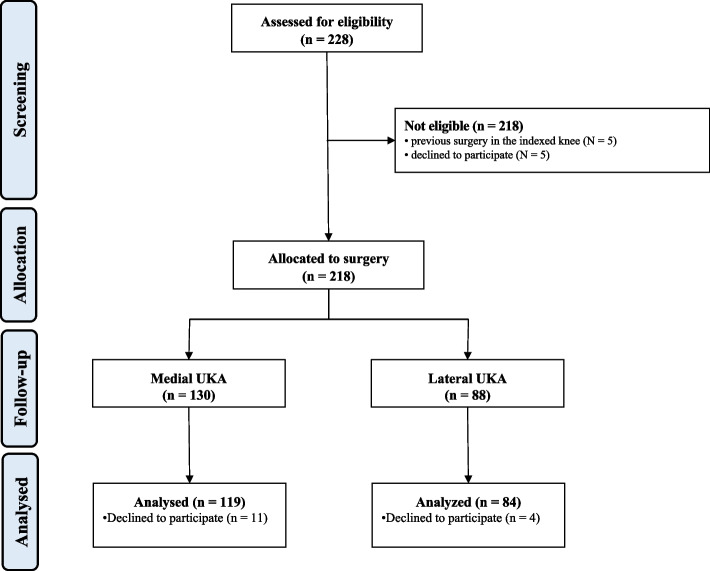
STROBE flow chart

Initially, 228 patients were recruited. Of them, five patients (*n* = 3 anterior cruciate ligament reconstruction, *n* = 2 tibial osteotomy) were excluded as they have undergone previous surgery on the knee. A further five patients declined to participate. A total of 223 patients underwent surgery. Of them, 15 patients were lost at follow-up. This left 203 patients for study: 119 patients were included in the medial UKA and 84 in the lateral cohort (Fig. 1).

### Patient demographic

The mean age of the patients was 68.9 ± 6.7 years and the mean BMI was 28.1 ± 4.1 kg/m^2^. The mean OKS on admission was 22.1 ± 4.5 points. The lateral group showed greater OKS on admission; comparability was found in mean age and BMI, ratio women:men, and length of the follow-up (Table 1).

**Table 1:** Demographic data and baseline comparability

Endpoint	Medial (*N* = 119)	Lateral (*N* = 84)	*P*
Age	69.7 ± 7.3	67.9 ± 5.8	0.07
BMI	27.9 ± 4.3	27.9 ± 4.0	0.9
Women	65 (54.6%)	45 (53.6%)	0.99
Follow-up (months)	26.9 ± 1.8	26.7 ± 1.7	0.4
OKS	21.3 ± 5.3	23.3 ± 2.5	0.03

### Results syntheses

At the last follow-up, no between groups difference was evidenced in OKS (Table 2). No patients experienced complications.

**Table 2:** Results of OKS at the last follow-up

Endpoint	Medial UKA (*N* = 119)	Lateral UKA (*N* = 84)	MD	SE	95% CI	*P*
OKS	44.5 ± 3.5	44.5 ± 1.7	0.0	0.41	-0.81 to 0.81	0.9

### Subgroup analysis

On admission, women, patients older than 70 years, and those with BMI lower than 28 kg/m^2^ who underwent lateral UKA evidenced lower OKS. At the last follow-up, no between groups difference was evidenced in all subgroups in OKS (Table 2).

## Discussion

The results of the present study confirmed our hypothesis that medial and lateral UKA achieve similar OKS at a minimum of two years of follow-up.

Both implants were associated with an improvement in the OKS. The OKS at baseline was greater in patients who have undergone lateral UKA. In addition, the medial group evidenced greater OKA in the subgroups women, age greater than 70 years, and BMI greater than 28 kg/m^2^ at baseline. Despite these differences, the same endpoints evidenced similar OKS at the last follow-up indicating that UKA improved OKS irrespective of the side, or minimal differences in sex, age, and BMI pre-operatively (Table 3).

**Table 3:** Subgroup analyses

Endpoint	Medial UKA	Lateral UKA	*P*
**Women**	***N* = 45**	***N* = 65**	
OKS Pre op	23.60 ± 2.51	21.14 ± 5.28	0.02
OKS post op	44.62 ± 1.68	44.17 ± 3.68	0.9
**Male**	***N* = 39**	***N *= 54**	
OKS Pre op	23.03 ± 2.48	21.48 ± 5.38	0.06
OKS post op	44.41 ± 1.73	44.89 ± 3.20	0.05
**Age < 70 years**	***N* = 40**	***N* = 51**	
OKS Pre op	23.65 ± 2.50	22.49 ± 4.15	0.1
OKS post op	44.65 ± 1.70	44.63 ± 2.99	0.4
**Age ≥ 70 years**	***N* = 44**	***N* = 68**	
OKS Pre op	23.05 ± 2.49	20.40 ± 5.91	0.02
OKS post op	44.41 ± 1.70	44.40 ± 3.81	0.2
**BMI < 28 kg/m**^**2**^	***N* = 44**	***N* = 68**	
OKS Pre op	23.48 ± 2.54	20.32 ± 5.16	0.001
OKS post op	44.39 ± 1.71	45.13 ± 2.90	0.05
**BMI ≥ 28 kg/m**^**2**^	***N* = 40**	***N* = 51**	
OKS Pre op	23.18 ± 2.47	22.59 ± 5.27	0.6
OKS post op	44.67 ± 1.69	43.65 ± 3.99	0.6

To the best of our knowledge, a formal minimal clinically important difference (MCID) for the OKS in UKA has not been established. According to previous studies on primary or revision TKA, the MCID of the OKS was 5% [8, 22]. Considering our results, the OKS of both groups improved more than 20% at the last follow-up, which is well beyond its MCID. Previous studies found similar improvements in the OKS. Baur et al. [4] reported a median improvement of the OKS of 43% at approximately three years of follow-up. Baryeh et al. [3] reported a median OKS of 43 on 898 patients who underwent UKA at two years of follow-up. These results are supported also by a recent meta-analysis of 47 studies (2,651 patients) on lateral UKA reporting a mean improvement of the OKS of 17.5% (range, 12.7 to 25.7) [6]. Similar findings were evidenced in another systematic review of four studies (*n* = 3,417) at a mean of 10 years of follow-up [40] and comparing the OKS in robotic-assisted and manual UKA [15].

Given the higher rate of medial osteoarthritis, lateral UKAs are less commonly performed. Indeed, medial UKAs are performed approximately ten times more frequently than lateral UKAs. Compared to the medial UKA, there is a paucity of evidence on lateral UKA in the current literature. Differences in anatomy and biomechanics between the two compartments should be considered. Given the convexity of the lateral tibial plateau and the C-shaped lateral meniscus, the lateral compartment has greater mobility [55]. Additionally, the screw-home mechanism and femoral rollback are also more pronounced at the lateral side [26, 55]. This tendency was also evident in the first mobile-bearing UKA implants, where the flat tibial component laterally increased the likelihood of bearing dislocation [7]. Given the higher complexity of lateral compartment biomechanics and the paucity of studies examining lateral UKAs designs and positioning, medial implants have historically been thought to be at a lesser risk of failure [16].

At a minimum follow-up of five years, no difference in medial and lateral UKA in the revision rate and implant survivorship was observed in 223 patients [18]. We could not identify previous studies that have compared medial and lateral UKA in OKS. Previous authors referred to the Knee Society Score (KSS), Forgotten Joint Score (FJS), Knee Injury and Osteoarthritis Outcome Score (KOOS), 12-Item Short Form (SF-12), and Western Ontario and McMaster Universities Arthritis Index (WOMAC). Despite the different PROMs used, there was consensus that medial and lateral UKA achieved similar clinical and functional outcomes at short- to midterm follow-up [2, 18, 29, 32, 37, 40, 52, 58].

The evidence on lateral fixed-bearing Zuk (Lima Corporate, Udine, Italy) implants is limited. For the medial component, fixed-bearing medial PPK (Zimmer-Biomet, Warsaw, Indiana, USA) was used. This implant has been already evaluated in a previous clinical trial of the same group, with similar improvement in the OKS at a 3-year follow-up [10]. The same implant was used in another study on 460 patients [56]. Similar to the present study, at approximately five years of follow-up, the mean OKS was 43.3 [56].

UKA restores the joint line to its native level and recreates the natural slope [56]. Medial and lateral OA patterns are different, although they are characterised by the same degeneration process. During the motion, there are higher degrees of translation and rotation of the lateral femoral condyle on the lateral tibia, which increases pain during the flexion in the lateral compartment [5, 46]. On the contrary, in the medial compartment, the middle and anterior aspects of articular cartilage are most commonly degenerated, which causes pain during extension [54].

All operations have been performed by a single surgeon well beyond the learning curve in a single centre; therefore, the number of procedures studied is limited. By definition, this study cannot be randomised, as we compared different aetiologies and surgical indications. The risk of performance bias was high since patients were unblinded to the procedure. Blinding in elective orthopaedic surgery is difficult to conduct. Future investigations are required to establish whether medial and lateral UKA have different survivorship or progression of osteoarthritis progression patterns. Data on weight-bearing radiographs of the lower limb were not prospectively collected. These data could give information on the biomechanical axes of the lower leg, and open new insights on the comparison of medial and lateral UKA.

## Conclusion

Medial and lateral UKA achieve similar outcomes in OKS at a minimum of two years of follow-up.

## Data Availability

All data and materials are available on reasonable request to Dr. Riccardo D’Ambrosi (riccardo.dambrosi@hotmail.it).

## Abbreviations

TKA: Total knee arthroplasty
UKA: Unicompartmental knee arthroplasty
BMI: Body mass index
OKS: Oxford Knee Score
MRI: Magnetic resonance imaging
ROM: Range of motion
KSS: Knee Society Score
FJS: Forgotten Joint Score
KOOS: Knee Injury and Osteoarthritis Outcome Score
SF-12: 12-Item Short Form
WOMAC: Western Ontario and McMaster Universities Arthritis Index
STROBE: Strengthening the Reporting of Observational Studies in Epidemiology
